# Bendamustine, pomalidomide, and dexamethasone for relapsed and/or refractory multiple myeloma

**DOI:** 10.1038/s41408-018-0104-5

**Published:** 2018-07-31

**Authors:** Dharshan Sivaraj, Michael M Green, Yubin Kang, Gwynn D Long, David A Rizzieri, Zhiguo Li, Anderson H Garrett, Jackie L McIntyre, Nelson J Chao, Cristina Gasparetto

**Affiliations:** 1 Division of Cellular Therapy, Duke University, 2400 Pratt Street, Durham, NC 27708 USA; 2 Department of Biostatistics and Bioinformatics, 2424 Erwin Road Suite 1102 11086 Hock Plaza, Durham, NC 27705 USA

LETTER TO THE EDITOR

Innovative treatment strategies that are efficacious and tolerable are needed for patients with relapsed/refractory multiple myeloma (RRMM), particularly for those who have exhausted available treatment options. Pomalidomide is a potent immunomodulatory drug (IMiD^®^) agent with anti-angiogenic, anti-proliferative, and immunomodulatory activity against MM^1–3^. The combination of pomalidomide and dexamethasone (Pd) in patients with RRMM was studied in a phase III trial (MM-003) and showed a promising overall response rate (ORR: 31%) with an improvement in progression-free survival (PFS) and overall survival (OS). Bendamustine is a bifunctional mechlorethamine agent with preclinical activity in cell lines resistant to alkylators as well as clinical activity in patients with disease resistant to other alkylating agents^4–6^. Single-agent activity of bendamustine was evaluated in a phase I dose escalation trial conducted in patients with progressive disease after autologous stem cell transplantation (ASCT) and showed a robust ORR and promising potential for bendamustine to be used in combination with other anti-myeloma therapies^7^. Clinical experience supports the combination of bendamustine with the IMiD^®^ agents thalidomide and lenalidomide, as well as the proteasome inhibitors bortezomib and carfilzomib^8–11^. Of note, a phase I/II open-label study investigating the combination of bendamustine, lenalidomide, and dexamethasone (BLD) reported promising efficacy with tolerable side effects^12^. We hypothesized that the combination of bendamustine and pomalidomide would be an effective combination in RRMM, particularly in patients with alkylator refractory and lenalidomide refractory disease. Herein, we report the results of an open-label phase I/II dose-escalation trial (NCT01754402) of bendamustine, pomalidomide, and dexamethasone (BPD) in patients with RRMM.

Patients were required to have had a confirmed diagnosis of multiple myeloma that was relapsed after prior therapy or that was refractory to the most recently received therapy. All patients must have been pomalidomide naïve, have received prior lenalidomide, and have been determined to be refractory. Refractory was defined as a history of progression on a regimen containing full (25 mg) or maximally tolerated dose of lenalidomide administered for a minimum of at least two completed cycles of therapy. The study was approved by the institutional review board at Duke University and was conducted in accordance with the Declaration of Helsinki and the guidelines for good clinical practice.

This open-label, dose escalation study was performed in the United States at Duke University, Durham, NC. The phase I portion was designed to determine the maximum tolerated dose (MTD) (primary objective) of bendamustine and pomalidomide in combination with a fixed dose of dexamethasone for patients with relapsed or refractory MM. In the phase II portion of the study, an expansion cohort of patients was treated at the MTD to assess ORR. Secondary endpoints included evaluation of PFS, OS, and time to response. Individual patients stayed at the same dose level throughout the study unless they required dose reduction due to toxicity.

Patients were evaluated for dose-limiting toxicity (DLT) according to the National Cancer Institute Common Terminology Criteria for Adverse Events version 4.0. All patients were required to use deep vein thrombosis (DVT) prophylaxis, which was either aspirin (81 mg) daily or, for those with prior history of DVT, full anticoagulation treatment. Bendamustine was administered intravenously (IV) over 30 min on day 1 of cycle 1 for all cohorts at a starting dose of 120 mg/m^2^. Pomalidomide was administered orally once daily on days 1 to 21, every 28 days. Dexamethasone (40 mg) was administered weekly (oral or IV) on days 1, 8, 15, and 22, every 28 days (Supplementary Figure 1). This dosing strategy was designed to be more convenient for patients receiving therapy, as the infusions were administered just once per cycle. After the first six cycles, it was recommended that the dose of dexamethasone be reduced to 20 mg. Patients could proceed to the maintenance phase of the study after completing 12 cycles of treatment, during which they would discontinue bendamustine and continue with Pd until progression.

This study utilized a standard 3 + 3 dose-escalation schedule. All patients were considered evaluable for toxicity unless they could not complete the first cycle of therapy due to disease progression or withdrawal of consent. Patients must have completed two cycles of therapy to be evaluable for efficacy unless a patient was removed from the study before completing two cycles due to disease progression, although they would still be considered evaluable for response.

A total of 38 patients were enrolled at Duke University Medical Center in the United States between January 2013 and September 2016. We enrolled eight patients into the phase I dose escalation portion and 30 patients in the phase II dose expansion portion of the study. The median number of prior regimens was 5 (range, 3–8). In all, 100% (*n* = 38) of patients were refractory to full-dose lenalidomide and had received prior treatment with bortezomib. Thirty-one (82%) patients had undergone prior ASCT. Twelve (32%) patients had received prior carfilzomib. All patients were pomalidomide naive (Table 1). All patients were included in the intent-to-treat response evaluation.

**Table 1:** Baseline patient demographics, disease, and treatment characteristics

	*N* = 38
Median age, median (range), years	67 (47–83)
**Sex, no. (%)**	
Male	17 (45)
Female	21 (55)
ECOG performance status, no. (%)	
0	7 (18)
1	25 (66)
2	6 (16)
Median time since initial diagnosis, y (range)	3.6 (.75-9.86)
Prior regimens, median (range)	5 (3-8)
**Prior therapies, no. (%)**	
Transplant	31 (82)
Bortezomib	38 (100)
Lenalidomide	38 (100, all refractory)
Carfilzomib	12 (32)
**Cytogenetics**	
Hypodiploid	1
Hyperdiploid	12
Del(13)	7
Del(17p)	6
t(4;14)	4
t(11;14)	7
+ 1q	4

*ECOG* Eastern Cooperative Oncology Group

In the first dosing cohort (bendamustine 120 mg/m^2^, pomalidomide 3 mg, and dexamethasone 40 mg), one of six patients encountered a protocol-defined DLT of nausea, diarrhea, and vomiting. At the second-dose level (bendamustine 120 mg/m^2^, pomalidomide 4 mg, and dexamethasone 40 mg), the first two enrolled patients experienced a DLT as a result of the study treatment. The first patient experienced grade 4 rash and the second patient experienced grade 3 febrile neutropenia. As a result of these two DLTs in cohort 2, the MTD was determined to be dose level 1 (bendamustine 120 mg/m^2^, pomalidomide 3 mg, and dexamethasone 40 mg) (Supplementary Table 1). Thirty additional patients were enrolled at the MTD in the dose-expansion phase, in order to assess the preliminary activity of BPD and further establish the safety profile.

The most common grade 3 or greater adverse events (AEs) included neutropenia (47%), anemia (26%), thrombocytopenia (21%), leukopenia (18%), and lymphopenia (24%). Alongside the high frequency of neutropenia, we observed five patients (13%) with grade 3–4 febrile neutropenia. Infections included five patients with grade 3 pneumonia, one patient with grade 4 pneumonia, and two patients with grade 4 sepsis. Of the 38 patients evaluable for toxicity, 29 (76%) had a grade ≥ 3 AE related to the study treatment. A total of 18 of 38 patients (47%) experienced grade 4 AEs. Hematologic and non-hematologic toxicities are listed in Supplementary Table 2. Grade ≥ 3 hematologic toxicities were prevalent but effectively managed via dose reductions and supportive care. The majority of patients discontinued therapy for disease progression or lack of response, as well as due to toxicities including pancytopenia, neutropenia, thrombocytopenia, nausea, fever, rash, and diarrhea. There were no deaths directly attributable to the study treatment and no incidences of secondary malignancies. The incidence of hematologic and non-hematologic AEs were similar to those reported by Lentzsch et al.^12^, in their phase I/II open-label, dose-escalation trial of BLD in RRMM.

All 38 patients were included in the intent-to-treat response assessment. Patients evaluable for efficacy received a median of 7 cycles (range, 1–31) of BPD. The ORR was 61% and the clinical benefit rate (CBR) ( ≥ MR) was 63% with 3 of 38 patients (8%) achieving a stringent complete response (sCR), 3 of 38 patients (8%) achieving a very good partial response (VGPR), 17 of 38 patients (45%) achieving partial response (PR), 1 of 38 patients (3%) achieving minimal response (MR), and 12 of 38 patients (32%) with stable disease (SD). Seven (18%) patients initiated the maintenance phase in cycle 13. After a median follow-up of 17.5 months (range, 2.4–31.6), the median PFS is 9.6 months (95% confidence interval (CI), 6.8–18.0), and the median OS is 21.3 months (95% CI, 12.3–N/A), with 24 patients (63%) surviving ≥ 12 months (Fig. 1). The efficacy of this regimen was observed in all cytogenetic subgroups with no significant difference in PFS and OS between those with intermediate-/high-risk disease and those with standard risk disease based on cytogenetic data at diagnosis (Supplementary Table 3, Supplementary Figure 2).

**Fig. 1:**
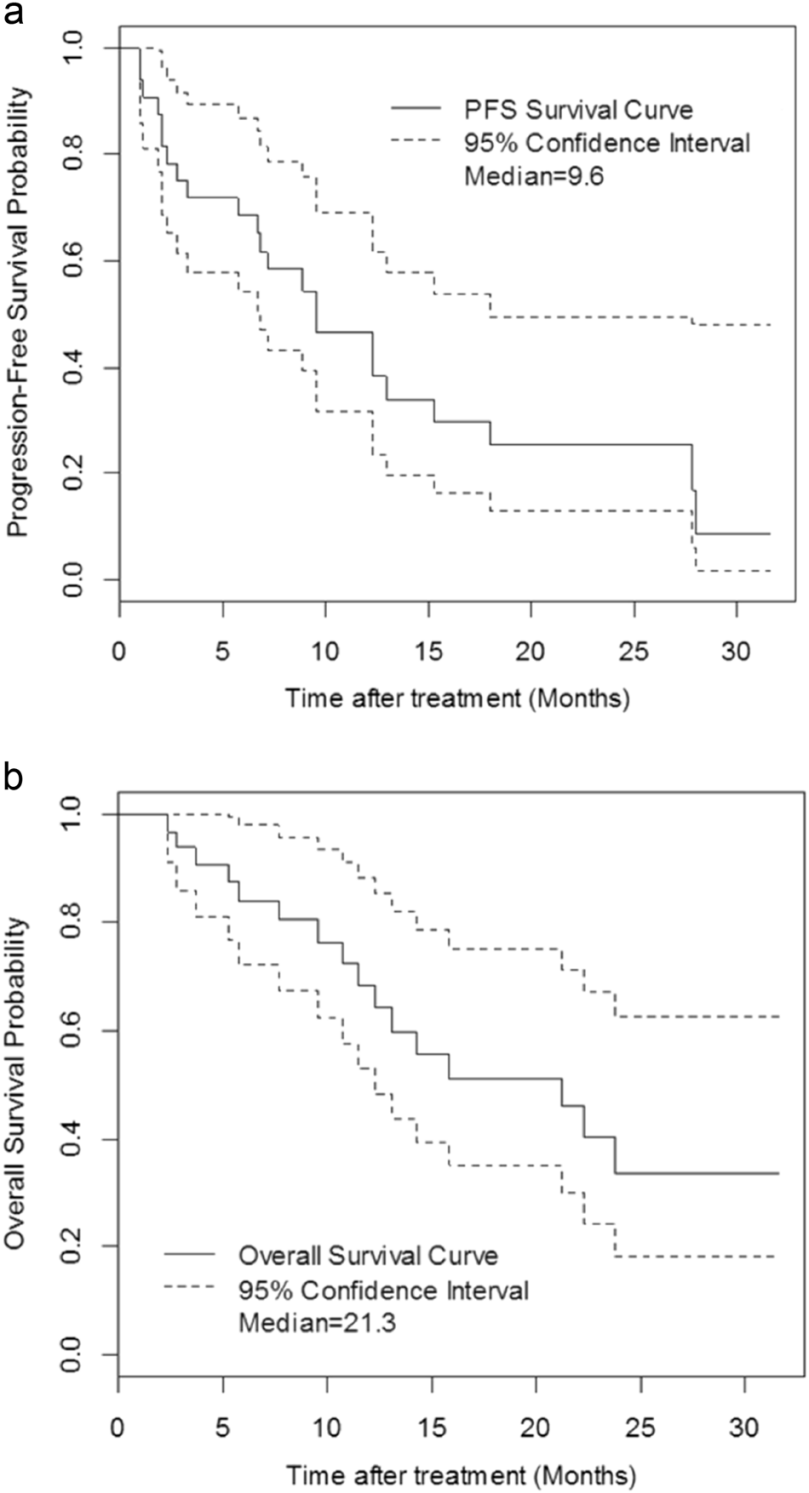
Progression-free survival and overall survival. Kaplan–Meier estimates (in months) of **a** PFS and **b** OS in evaluable patients treated with BPD for RRMM

We observed a promising ORR (61%) despite enrolling a patient population that was uniformly refractory to full-dose lenalidomide with prior bortezomib exposure, and 31% of patients having received prior therapy with carfilzomib. Our ORR compares favorably with other single-arm triplet combinations involving Pd in RRMM such as carfilzomib/pomalidomide/dexamethasone, and daratumumab/pomalidomide/dexamethasone, which have displayed ORRs of 55% and 60%, respectively^13,14^. The combination of bendamustine 120 mg/m^2^, pomalidomide 3 mg, and dexamethasone 40 mg is feasible and active in patients with heavily pretreated RRMM.

## Electronic supplementary material

Supplementary Figure 1

Supplementary Figure 2

Supplementary Table 1

Supplementary Table 2

Supplementary Table 3
