# Supplementary material for: Bendamustine, pomalidomide, and dexamethasone for relapsed and/or refractory multiple myeloma
Source: Blood Cancer J. 2018 Jul 31;8(8):71. doi: 10.1038/s41408-018-0104-5 (PMC6068091; doi:10.1038/s41408-018-0104-5)
Supplement: Supplementary file 1 — Supplementary Figure 1 [file 41408_2018_104_MOESM1_ESM.docx]

**Supplementary Figure 1. Treatment Schema**

**Days of cycle**

**Bendamustine**

Doses according to cohort

| 1 |  |  |  |  |  |  |  |  |  |  |  |  |  |  |  |  |  |  |  |  |  |  |  |  |  |  |  |  |  |
| --- | --- | --- | --- | --- | --- | --- | --- | --- | --- | --- | --- | --- | --- | --- | --- | --- | --- | --- | --- | --- | --- | --- | --- | --- | --- | --- | --- | --- | --- |

**Pomalidomide**

Doses according to cohort

| 1 21 |  |  |  |  |  |  |  |  |  |
| --- | --- | --- | --- | --- | --- | --- | --- | --- | --- |

**Dexamethasone**

Cycles 1-6: 40mg

Cycles >6: 20mg

| 1 |  |  |  |  |  |  | 8 |  |  |  |  |  |  | 15 |  |  |  |  |  |  | 22 |  |  |  |  |  |  |  |  |
| --- | --- | --- | --- | --- | --- | --- | --- | --- | --- | --- | --- | --- | --- | --- | --- | --- | --- | --- | --- | --- | --- | --- | --- | --- | --- | --- | --- | --- | --- |
