# Supplementary material for: Bendamustine, pomalidomide, and dexamethasone for relapsed and/or refractory multiple myeloma
Source: Blood Cancer J. 2018 Jul 31;8(8):71. doi: 10.1038/s41408-018-0104-5 (PMC6068091; doi:10.1038/s41408-018-0104-5)

**Supplementary Figure 2. Progression free survival and overall survival by genetic risk classification. Kaplan-Meier estimates (in months) of (A) PFS and (B) OS in evaluable patients treated with BPD for RRMM.**


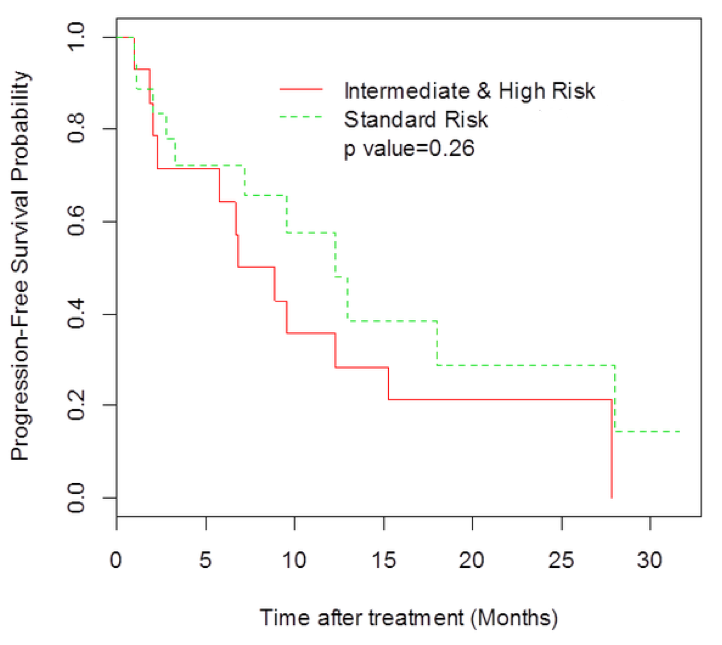


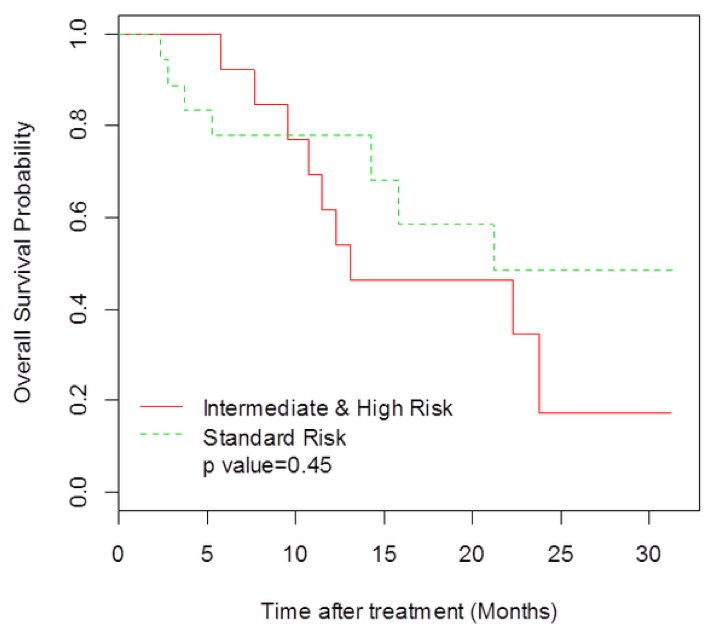

Supplement: Supplementary file 2 — Supplementary Figure 2 [file 41408_2018_104_MOESM2_ESM.docx]
