# Supplementary material for: Bendamustine, pomalidomide, and dexamethasone for relapsed and/or refractory multiple myeloma
Source: Blood Cancer J. 2018 Jul 31;8(8):71. doi: 10.1038/s41408-018-0104-5 (PMC6068091; doi:10.1038/s41408-018-0104-5)
Supplement: Supplementary file 3 — Supplementary Table 1 [file 41408_2018_104_MOESM3_ESM.docx]

**Supplementary Table 1. Planned dosing cohorts**

| **Cohort** | **Bendamustine, mg/m^2^** | **Pomalidomide, mg** | **Dexamethasone*, mg** |
| --- | --- | --- | --- |
| -1 | 120 | 2 | 40 |
| 1 (initial dose level) | 120 | 3 | 40 |
| 2 | 120 | 4 | 40 |
| 3 | 150 | 4 | 40 |
| 4 | 180 | 4 | 40 |

Bendamustine was administered IV over 30 minutes; pomalidomide was administered orally; dexamethasone was administered either IV or orally.

*Dosing of dexamethasone may be adjusted at any point during the trial at the discretion of the PI
