# Supplementary material for: Bendamustine, pomalidomide, and dexamethasone for relapsed and/or refractory multiple myeloma
Source: Blood Cancer J. 2018 Jul 31;8(8):71. doi: 10.1038/s41408-018-0104-5 (PMC6068091; doi:10.1038/s41408-018-0104-5)
Supplement: Supplementary file 4 — Supplementary Table 2 [file 41408_2018_104_MOESM4_ESM.docx]

**Supplementary Table 2. Common Adverse events (>25%), by maximum grade reported**

|  | **Grade** | | | | **Total patients, N (%) (N=38)** |
| --- | --- | --- | --- | --- | --- |
|  | **1** | **2** | **3** | **4** |  |
| **Hematologic AEs, n** |  |  |  |  |  |
| Anemia | 6 | 11 | 10 | 0 | 27 (71) |
| Thrombocytopenia | 9 | 4 | 4 | 4 | 21 (55) |
| Neutropenia | 6 | 5 | 7 | 11 | 29 (76) |
| Febrile Neutropenia | 1 | 0 | 5 | 0 | 6 (16) |
| Leukopenia | 0 | 8 | 5 | 2 | 15 (40) |
| Lymphopenia | 1 | 0 | 4 | 5 | 10 (26) |
| **Nonhematologic AEs, n** |  |  |  |  |  |
| Diarrhea | 5 | 2 | 3 | 0 | 10 (26) |
| Nausea | 8 | 1 | 3 | 0 | 13 (34) |
| Fatigue | 5 | 12 | 3 | 0 | 20 (53) |
| Dyspnea | 9 | 3 | 3 | 0 | 15 (39) |
| Rash | 5 | 3 | 1 | 2 | 11 (29) |
| Hypomagnesemia | 10 | 0 | 1 | 0 | 11 (29) |
| Hypokalemia | 11 | 1 | 2 | 1 | 15 (40) |
| Hypocalcemia | 12 | 1 | 1 | 0 | 14 (37) |
| Fever | 9 | 3 | 1 | 0 | 13 (34) |
