# Supplementary material for: Bendamustine, pomalidomide, and dexamethasone for relapsed and/or refractory multiple myeloma
Source: Blood Cancer J. 2018 Jul 31;8(8):71. doi: 10.1038/s41408-018-0104-5 (PMC6068091; doi:10.1038/s41408-018-0104-5)
Supplement: Supplementary file 5 — Supplementary Table 3 [file 41408_2018_104_MOESM5_ESM.docx]

**Supplementary Table 3. Response among evaluable patients and selected cytogenetic subgroups**

| **Response category, n (%)** | **All evaluable patients,**  **N = 38** | **Hyperdiploid,**  **n = 10** | **Del(13),**  **n = 5** | **Del(17p),**  **n = 6** | **t(11;14),**  **n = 4** | **+1q,**  **n = 4** | **t(4;14), n = 4** |
| --- | --- | --- | --- | --- | --- | --- | --- |
| **ORR** | 23 (61) | 6 (60) | 2 (40) | 4 (67) | 3 (75) | 3 (75) | 2 (50) |
| **sCR** | 3 (8) | 0 | 1 (20) | 1 (17) | 0 | 0 | 2 (50) |
| **VGPR** | 3 (8) | 2 (20) | 0 | 0 | 1 (25) | 0 | 0 |
| **PR** | 17 (45) | 4 (40) | 2 (40) | 3 (50) | 2 (50) | 3 (75) | 0 |
| **MR** | 1 (3) | 0 | 0 | 0 | 0 | 0 | 0 |
| **SD** | 12 (32) | 2 (20) | 2 (40) | 2 (33) | 1 (25) | 1 (25) | 2 (50) |
| **PD** | 2 (5) | 2 (20) | 0 | 0 | 0 | 0 | 0 |
